# Green nanocoating-based polysaccharides decorated with ZnONPs doped Egyptian kaolinite for antimicrobial coating paper

**DOI:** 10.1038/s41598-023-38467-1

**Published:** 2023-07-15

**Authors:** Mohamed S. Hasanin, Houssni El Saied, Fatma A. Morsy, Hanaa Hassan Abdel Latif Rokbaa

**Affiliations:** 1 Cellulose and Paper Department, National Research Centre, Dokki, 12622 Cairo Egypt; 2 Paper and Printing Lab., Chemistry Department, Faculty of Science, Helwan University, Helwan, Egypt

**Keywords:** Chemistry, Materials science

## Abstract

Paper coating plays an important role in the paper properties, printability and application. The nanocoating is a multifunction layer that provides the paper with unique features. In this work, nanocoating formulas were prepared using a green method and component. The nanocoating formulas were based on biopolymers nanostarch NSt and nanochitosan NCh (NCS) decorated with Egyptian kaolinite Ka doped with zinc nanoparticles NCS@xka/ZnONPs (x represents different ratios) support for multifunctional uses. The nanocoating formulas were characterized using a physiochemical analysis as well as a topographical study. FTIR, XRD, SEM and TEM techniques were used. Additionally, the antimicrobial activity of the tested samples was assessed against six microorganisms including Gram-negative and Gram-positive bacteria. The prepared nanocoating formulas affirmed excellent antimicrobial activity as a broad-spectrum antimicrobial active agent with excellent activity against all representative microbial communities. The nanocoating with the highest ratio of Ka/ZnONPs (NCS@40 ka/ZnONPs) showed excellent antimicrobial activity with an inhibition percentage of more than 70% versus all microorganisms presented. The paper was coated with the prepared suspensions and characterized concerning optical, mechanical and physical properties. When Ka/ZnONPs were loaded into NCS in a variety of ratios, the characteristics of coated paper were enhanced compared to blank paper. The sample NCS@40 ka/ZnONPs increased tensile strength by 11%, reduced light scattering by 12%, and improved brightness and whiteness by 1%. Paper coated with NCh suspension had 35.32% less roughness and 188.6% less porosity. When coated with the sample NCS@10 ka/ZnONPs, the coated paper's porosity was reduced by 94% and its roughness was reduced by 10.85%. The greatest reduction in water absorptivity was attained by coating with the same sample, with a reduction percentage of 132%.

## Introduction

Paper is the first material that is used on the earth as a multipurpose for various purposes such as writing, printing and conventional packaging^1,2^. Regrettably, today the neat paper sheets are not fitting enough as the case in the past. The coated paper consists of a base paper and a coating layer as well as it is an excellent technique for enhancing the visual and printing qualities of paper^1^. Coated paper is more uniform and more receptive to ink, resulting in superior printing results^3^. The primary components of coating color are pigments, binders, and various additives. Pigment characteristics, including particle size, size distribution, and morphology, play a crucial role in determining coated paper quality^4^. After pigments, binders are the second most abundant component in coating color^5^. Environmental issues are a major concern for humans and other organisms today.

Many synthetic products and materials have been developed over time to meet human needs. These synthetic materials could have hazardous effects on the environment and life^6^. During the past decades, numerous efforts have been made to develop natural or synthetic binders that are inexpensive and environmentally friendly as treatment agents^7^. The binders are either natural polymers, like starch, chitosan and protein, or synthetic polymers, like styrene-butadiene, poly (vinyl acetate), and polyacrylates. Starch is an inexpensive and biodegradable biopolymer compared to synthetic latex^8,9^. The addition of modified starch can modify the rheology, prevent pigment flocculation, and enhance water retention^5^. Additionally, chitosan (Ch) is another abundant natural polysaccharide biopolymer that is attracting more interest due to its unique properties, including low cost, biological activity, nontoxicity, biodegradability, antioxidant, antibiofilm, and antimicrobial activities^10–12^. Antibacterial and antibiofilm properties of Ch are caused by its positively charged amino groups, which react with the bacterial membrane. Furthermore, Ch with large surface areas modifies the permeability of bacterial membranes through membrane incorporation, resulting in bacterial death^13–15^. However, the low mechanical properties of Ch during swelling and reduced antibacterial activity at neutral pH restrict its practical applications^16,17^. Indeed, bacterial resistance to antibiotics is a result of pathogens' ability to adapt to different environmental conditions and develop self-defense mechanisms, such as biofilm formation^18,19^.

Numerous research groups have focused on developing novel antimicrobial agents and materials to protect human life from the harmful effects of microorganisms^20,21^. In this context, chelation between functional groups in Ch and metallic salt solutions or the formation of CS/inorganic composites can further enhance the activity. Li et al.^22^ successfully prepared composites from zinc, chitosan and sepiolite. Their findings demonstrated that smaller ZnO nanoparticles in CTs/ZnO-Sep may easily enter the bacterial cell membrane and cause cell damage, and a higher positive charge of CTs-ZnO in CTs/ZnO-Sep improved the interaction of CTs-ZnO with the cell membrane and so improved antibacterial capabilities. Due to the combined antibacterial activities of Ch and ZnO NPs, Ch-ZnO nanocomposites can be utilized as effective antibacterial and antibiofilm agents^7^. Due to its antibacterial and antibiofilm activities against a wide variety of bacteria, zinc oxide (ZnO) has received increased attention^23^. Chitosan and zinc oxide nanocomposites provide a new generation of biopolymer nanocomposites that are effective against microbial infections and environmental pollutants^24,25^. Incorporating inorganic nanomaterials into an organic polymer system produces new composite materials with enhanced properties and superior performance compared to the constituents^26,27^.

Kaolin is one of the most widely used pigments in the paper industry and has a wide variety of coating applications. The mineral kaolinite (Al_2_SiO_5_(OH)_4_) is the primary component of China clay or kaolin. Indeed, the Egyptian kaolinite is a abundant clay type in Egypt that characterized with economic and availability as well as suitability for server industrial applications as filler or coating^28–30^. The successful modification allowed for the use of Egyptian kaolinite as a pigment for paper coating^31,32^. The majority of coated paper's characteristics, as well as paper printability, were improved^33,34^. Due to their potent intercalation capacity, excellent thermal stability, and low cost, natural clay minerals are acknowledged as the ideal inorganic carriers in antibacterial materials^22^.

Therefore, the presented work aims to introduce green synthesis nanocoating based nanochitosan and nanostarch decorated with ZnONPs doped Egyptian kaolinite for paper coating for the first time. This formulated nanocoating was reinforced as a multifunction namely; excellent optical, mechanical and antimicrobial properties. Moreover, specially designed nanocoating was strengthened to have superior optical, mechanical, and antibacterial qualities.

## Experimental

### Martials

Zinc acetate penta hydrate was purchased from Loba chem., India. Chitosan (Ch) used in this study was purchased from Sigma-Aldrich (St. Louis, and molecular weight 650,000) and included low molar mass CS (LCS) (viscosity, 275.9 cps, degree of deacetylation, 85.5%) and reduced to nanosized as reported in our previous work^35^. Starch was extracted from potato peel waste as described in our previous work^36^. All chemical reagents and microbial media were used in analytical grade and purchased from Loba Chem., India. Throughout the experimental work, kaolinite pigment (Ka) of a particle size 2 µm (the main constituents (%): Al_2_O_3_ 35.051, SiO_2_ 44.609, TiO_2_ 3.531 and Fe_2_O_3_ 1.253) was used. It was supplied by Alarabiya Co. for trade and industry, in Egypt. An uncoated office paper with a grammage equal to 70 g/m^2^ was used.

### Preparation of green nanocoating

The green nanocoating was prepared using biopolymers including nanochitosan and nanostarch which were decorated with Ka/ZnONPs that were prepared via a green method as well.

#### Preparation of Ka/ZnONPs

Preparation of Ka/ZnONPs was carried out using microwave radiation and followed with ultrasonication. In particular, 1 g of kaolinite was dispersed in 10 mL deionized water and stirred at 1500 rpm at room temperature for 30 min. zinc acetate (0.1 g) was added stepwise at the same condition. After, the prepared solution temperature was raised to 70 °C for 5 h. This step was followed by microwaves (1000W) for 2 min and ultrasonication for 10 min.

#### Green nanocoating preparation

Separated solutions of nanochitosan and nanostarch (1% w/v) were prepared and mixed with stirring at 1500 rpm at 70 °C for 1 h then this sample was treated by ultrasonication for 1 min and called (NCS). The Ka/ZnONPs were loaded with different ratios into NCS as 10, 20, and 40% (v/v), and these samples were called NCS@10 ka/ZnONPs, NCS@20 ka/ZnONPs, and NCS@40 ka/ZnONPs, respectively.

### Preparation of coated paper samples

An automatic bar coater (K Control Coater, Model 101) was used for applying the coating mixtures. A wire-wound coating bar was chosen to give a 6 μm thick wet film. The selected office paper was cut to 19.6 × 29.6 cm^2^ using an “Ideal Strip” cutter. It was considered blank and coated with the prepared coating suspensions.

### Characterizations

#### Green nanocoating characterizations

The prepared samples were characterized, including topographical and physiochemical characterizations. The topographical study involved a High-resolution transmission electron microscope (HRTEM, JEOL 2010, Japan) operating at 300 kV. It was used to examine the particle shape and size of the prepared nanoparticles and select areas for electron diffraction (SAED). Scanning electron microscopy (SEM), Quanta FEG 250, FEI, Republic of Czech) linked with energy dispersive X-ray analysis (EDX; Model Quanta 250 FEG, Field Emission Gun) (of note this SEM was used for coating paper sheet). Additionally, the physiochemical characterizations were studied via Attenuated total reflection Fourier-transform infrared (ATR-FTIR) spectroscopy “Spectrum Two IR Spectrometer-PerkinElmer, Inc., Shelton, USA”. Spectral analysis was obtained at 32 scans and 4 cm^−1^ resolutions in wavenumbers ranging from 4000 to 400 cm^−1^. The x-ray diffraction (XRD) patterns of the different samples were investigated using a Diano X-ray diffractometer (Philips) provided with a Cu Kα radiation source (λ = 0.15418 nm), energized at 45 kV, as well as a generator (PW 1930) and a goniometer (PW 1820).

#### Coated paper samples characterization

The properties of coated paper samples, including the prepared suspensions, were evaluated using standard tests. Table 1 represents the coated paper properties, instruments, and the standard methods followed for coated paper characterization.

**Table 1:** Instruments and standard methods for coated paper properties.

Coated paper properties	Standard methods	Instruments
Optical properties		Brightness and Colormeter instrument, model 68-59-00-002, Buchel-B.V, Netherlands
Brightness %	ISO 2470–1 (2009)	
Whiteness%	ISO 11476 (2010)	
Opacity	ISO 2471 (2008)	
Light scattering Lu	ISO 9416 (2009)	
Mechanical properties		
Tensile strength (kN/mm)	ISO 1924-2 (2008)	Tensile Test machine, T-series; model H5KT, Tinius Olsen Ltd, at 1KN, UK
Tensile energy absorption (J/m^2^)		
Stretch (%)		
Physical properties		
Paper roughness ml/min	ISO 8791-2 (2013)	Bendtsen Tester, model 58-21-00-0002 (KS12), Messmer Buchel B.V., Netherlands
Air permeance µm/Pa S	ISO 5636 (2013)	
Cobb (water absorption)	ISO 535-1991(E)	Cobb water absorption tester, model P95.93000, FRANK-PTI, Germany

### Antimicrobial study

#### Microbial strains and growth conditions

The antimicrobial activity of tested samples was assessed against six microorganisms, including Gram-negative bacteria (*Escherichia coli* ATCC25922 & *Pseudomonas aeruginosa* ATCC 27853), Gram-positive bacteria (*Staphylococcus aureus* ATCC25923 & *Bacillus subtilis* ATCC6051), unicellular fungi (*Candida albicans* ATCC90028, and filamentous fungi (*Aspergillus niger* RCMB 02724). Bacterial strains were cultivated on nutrient broth at 37 °C for 24 h, while fungal strains were grown on malt extract broth medium then incubated for 3–5 days at 28 °C ± 2; and then kept at 4 °C for further use^37–40^. As mentioned in our previous work^41^, the cell formation unit counting (CFU) approach was applied. About 0.1 g of the sample weight was used. The antibiotics Streptomycin and Griseofulvin were used as standard a broad spectrum antibacterial and antifungal, respectively.

## Results and discussion

### Nanocoating characterizations

Characterizations of nanocoating formulas were included the physicochemical analysis to investigate the interaction between nanocoating complements as well as the behavior of Ka before and after being added into Ka/ZnONPs and other formulations. Moreover, the topographical study affirmed the surface texture and performance of the nanocoating that assist in the prediction of nanocoating role in improving the paper surface and other properties.

#### Topographical study of green nanocoating

**Figure 1:**
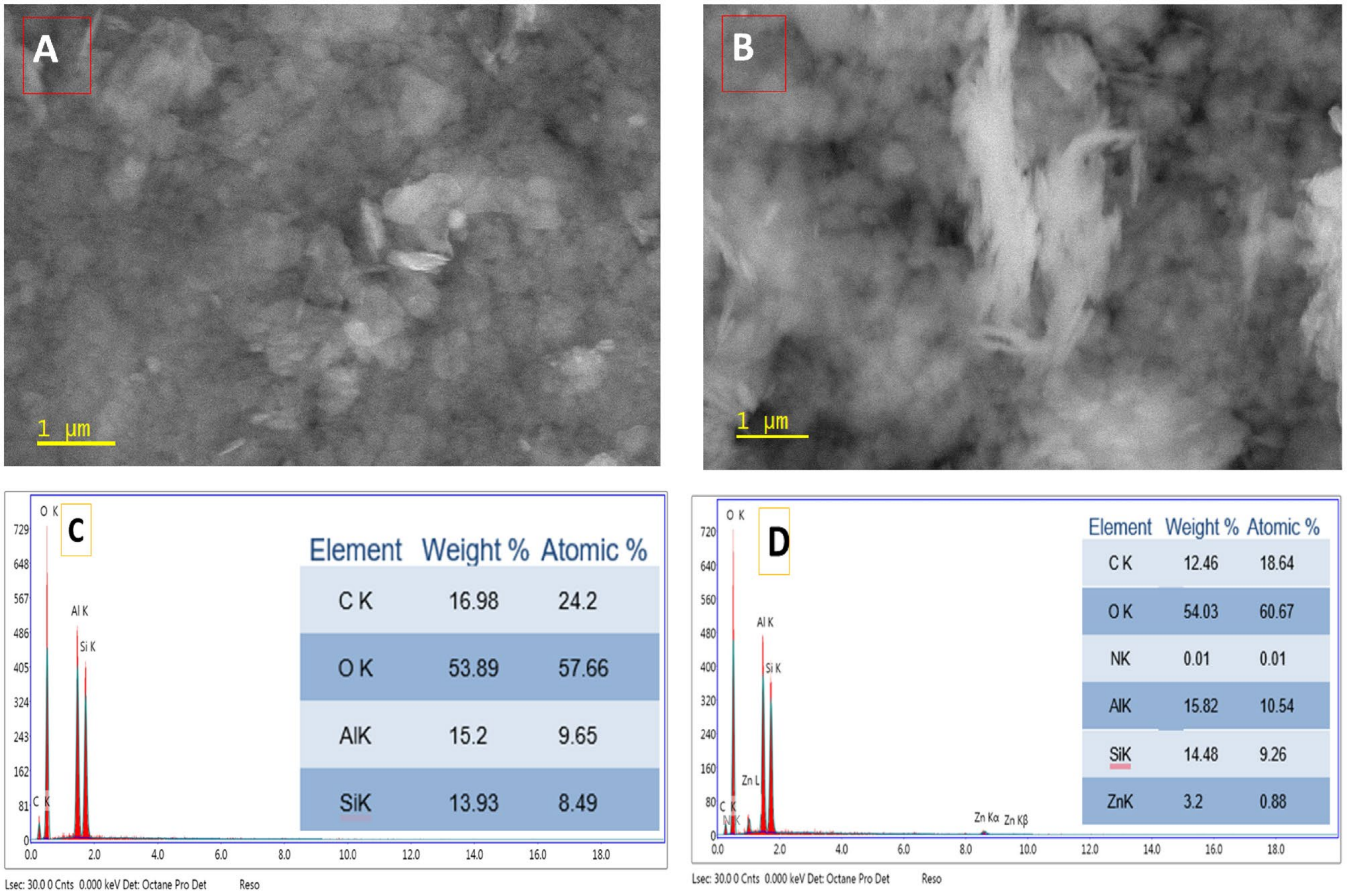
Topographical study included SEM images of kaolinite (**A**) and Ka/ZnONPs (**B**) as well as EDX charts of kaolinite (**C**) and Ka/ZnONPs (**D**).

The prepared green nanocoating was characterized topographically using SEM and EDX chart against the not modified kaolinite as shown in Fig. 1. The SEM image of kaolinite (Fig. 1A) was performed as a random particle with a uniform shape and the brightness is close to inorganic shine. Otherwise, the NCS@20 ka/ZnONPs were observed in Fig. 1B with more details in comparison with the blank kaolinite with clear rods for ZnONPs. In addition, the EDX chart of kaolinite (Fig. 1C) illustrated the atomic content as mainly Al and Si. This observation is fitted with kaolinite chemical composition. In this context, the EDX chart of NCS@20 ka/ZnONPs was assigned the same composition with the presence of Zn ion. These observations indicate that the doping process was carried out.

**Figure 2:**
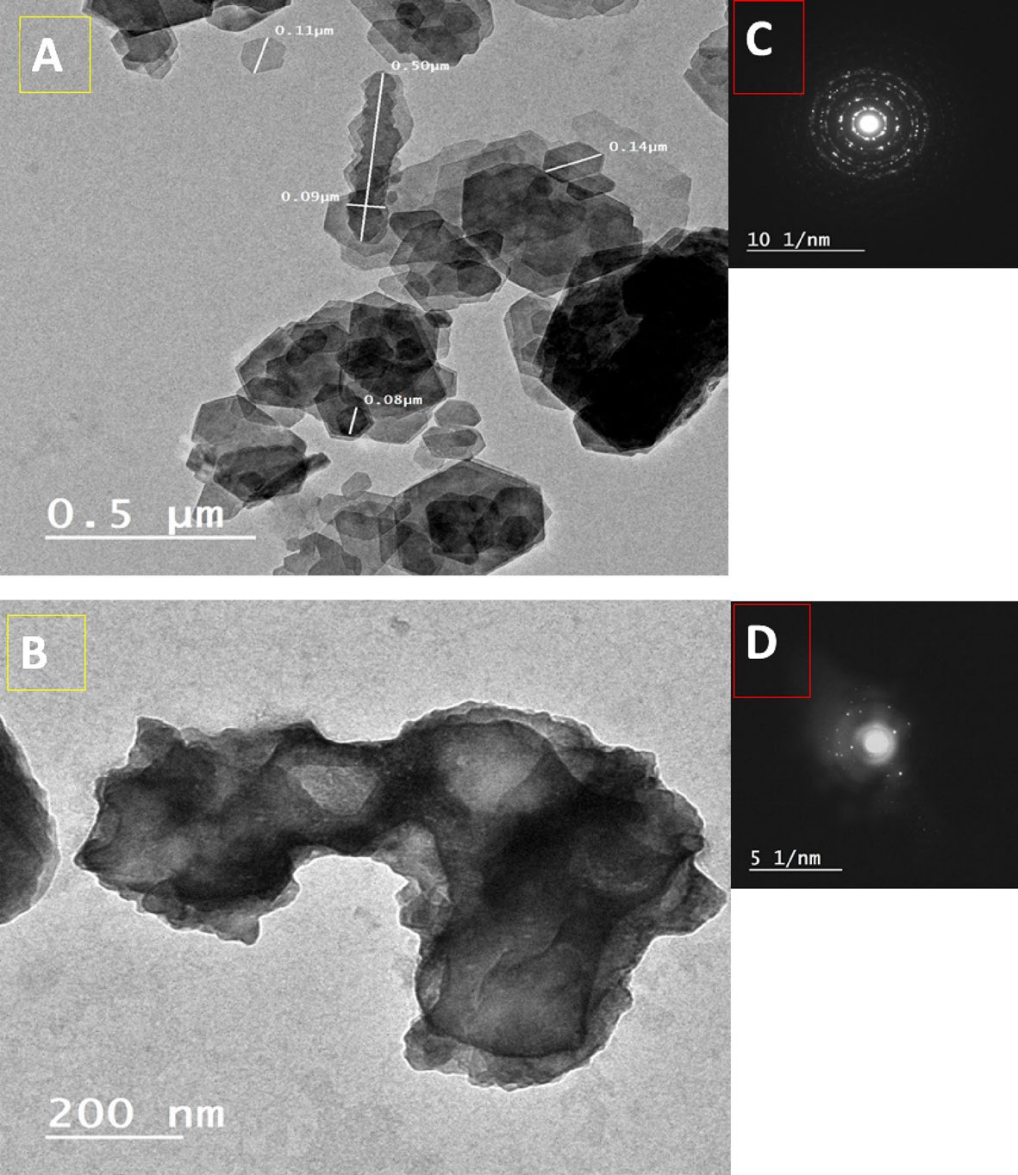
TEM image of Ka/ZnONPs (**A**) and NCS@20 ka/ZnONPs (**B**) as well as SEAD diffraction for Ka/ZnONPs (**C**) and NCS@20 ka/ZnONPs (**D**).

Nonetheless, Fig. 2 illustrated the TEM images and SEAD diffraction of Ka/ZnONPs and NCS@20 ka/ZnONPs. The Ka/ZnONPs TEM image (Fig. 2A) was shown as a plate structure aggregated with ZnONPs rods. Moreover, the SAED pattern of The Ka/ZnONPs (Fig. 2C) affirmed a high crystallinity behavior with rings arranged with sharp and shining spots distributed regularly on the rings. Likewise, the NCS@20 ka/ZnONPs TEM image was illustrated in Fig. 2B with a nice performance that related to the stabilization of the polysaccharide which fills the plats gabs of Ka/ZnONPs and appears as massive particles aggregated together. Furthermore, the SAED pattern of NCS@20 ka/ZnONPs (Fig. 2D) confirmed the image appearance, and the crystallinity performance was changed with a similar polycrystalline diffraction pattern. These observations affirmed that the polysaccharides affected the structure of Ka/ZnONPs that penetrate the plates form and stabilized the performance of the formulated structure.

#### Physiochemical characterizations

**Figure 3:**
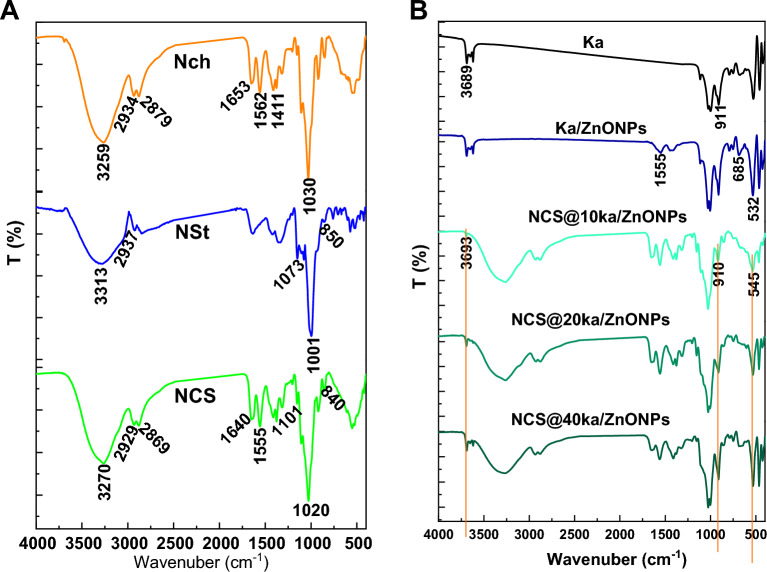
The FTIR spectra of nanocoating and their neat materials (**A**) and Ka and NCS@Ka/ZnONPs with different concentrations (**B**).

The physiochemical characterizations of the nanocoating neat component and different formulas of nanocoating were included in FTIR and XRD. The FTIR spectra were illustrated in Fig. 3. The nanocoating biopolymer components including NCh and NSt, as well as the NCS formula, were observed in Fig. 3A. NCh spectrum was observed, a characteristic band at 3259 cm^−1^ that corresponded to hydroxyl groups stretching. Additionally, the band of C–H stretching vibration was assigned as two small bands at 2934 and 2879 cm^−1^ due to the nanoformulation of chitosan^35^. Moreover, the bands at 1653, 1562, 1411, 1317 and 1030 cm^−1^ are attributed to C=O (stretch), N–H (bend), CH3 (stretch), C–N (stretch, –NHCO–CH3–) and C–O–C (glucos-amine ring), respectively^42^. Additionally, the NSt spectrum was shown as a characteristics bands at 3313, 2937, 1153, 1073, 1001 and 850 cm^−1^, which correspond to hydroxyl groups stretching, C–H stretching vibration, C–O–H groups, the C–O bond, the C–O–C group in the anhydroglucose rings and vibration of the C–O–C ring of starch, respectively^43,44^. On the other hand, the formulation of the Ch/St templet of nanocoating affects the FTIR spectrum, especially in both groups, namely, OH and CH. In particular, the OH band was observed to be sharper, as well as the frequency of the CH band, which was reduced to a lower position. This occurrence could be related to interactions between biopolymers. Furthermore, the Ka spectrum exhibited characteristic bands at 3689 and 911 cm^−1^ that were attributed to hydroxyl stretching bands to the inner surface, with hydroxyl groups oriented towards the vacant sites in the external layers of the kaolinite structure^34^. Otherwise, zinc oxide doping was observed in new bands at 1555, 685, and 532 cm^−1^ that were due to the incorporation of ZnO in the plating structure of Ka^45^. However, the incorporation of Ka/ZnONPs into nanocoating formulas with different concentrations that were assigned as new bands at 3693, 910, and 545 cm^−1^ was attributed to Ka, ZnO–Ka new bond and ZnONPs FTIR fingerprint region bands, respectively. Indeed, the intensity of these bands was going in a parallel direction with Ka/ZnONPs concentration.

**Figure 4:**
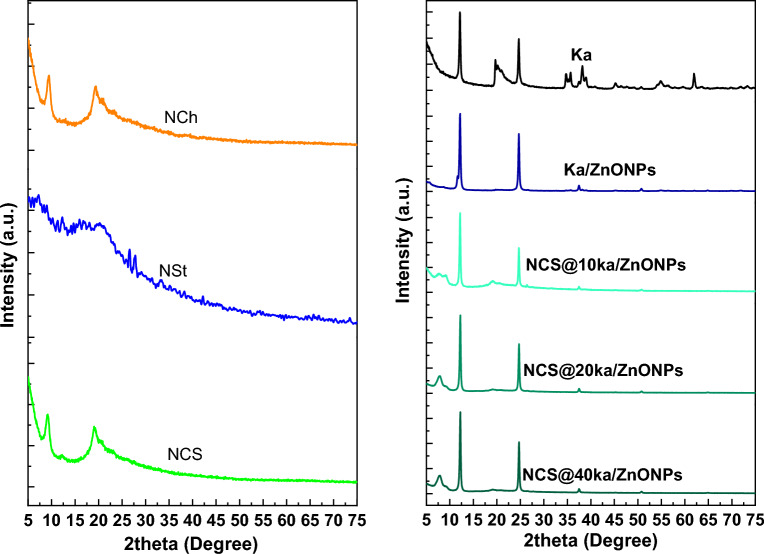
XRD patterns of nanocoating and their neat materials.

The crystallography study was shown in Fig. 4. The NCh crystallographic pattern was observed as a typical crystallography of NCh as reported in our previous work^14,46,47^ with high crystallinity and a sharp characteristic peak at around 20°^48^. In addition, the NSt pattern showed amorphous behavior with characteristic peaks at 16.9, 20.3, 26.6, and 27.8°^49,50^. On the other side, the NCS crystallographic pattern was observed with characteristics peaks at 19 and 23° which were related to neat materials peaks with slight shifting according to polysaccharide chain compensation. Moreover, the nanocoating loaded with Ka/ZnONPs with different ratios as well as the neat Ka and Ka/ZnONPs crystallography pattern was carried out. The Ka pattern detected sharp, obvious peaks at 12, 20, 35, 36, 39, 46, 55, and 61°, which are typical for the Egyptian Karoline structure^51,52^. Additionally, of ZnONPs effect on the Ka pattern, showed the disappearance of a peak at 20° as well as the appearance of small peaks at 38 and 51° that were due to the doping of ZnONPs into the Ka structure^53,54^. In addition, the different formulas of nanocoating patterns confirmed that the polysaccharide peak was around 10°. However, the crystallographic pattern of nanocoating formulas was similar due to the dominance of inorganic materials in the XRD pattern over polymers.

#### Paper coating topography

**Figure 5:**
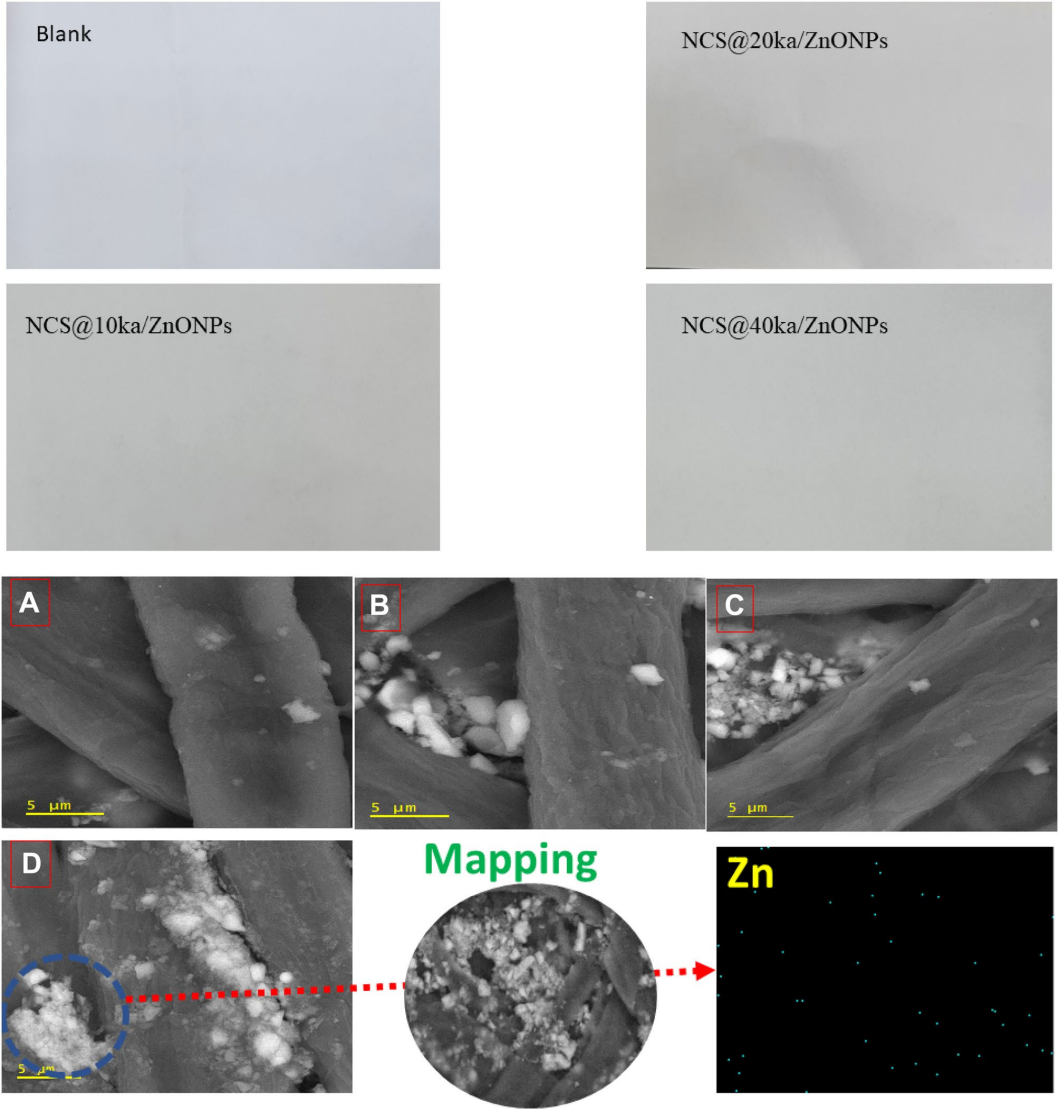
High resulsion photos of bank and coated paper sheets (upper) as well as the SEM images of blank paper (**A**), paper coated with NCS@10 ka/ZnONPs (**B**), NCS@20 ka/ZnONPs (**C**), and NCS@40 ka/ZnONPs (**D**) nanocomposites and mapping of the sample coated with NCS@40 ka/ZnONPs (lower).

Figure 5 (upper) showed the high resolution photos of coated paper sheets were comparison with the blank one. The black paper sheet was observed as a traditional paper sheets with some blueness color that reduced to yellowish white after coating with the different formulas. Moreover, the yellowing color was reduced to clear whit in sample NCS@40 ka/ZnONPs due clay and ZnoNPs white color. On the other side, the Fig. 5 (lower) was illustrated SEM images of the paper coated with different formulas of nanocoating compared with plank paper as well as mapping of the Zn ion of the highest formula Zn content. At first glance, the fibers of paper were observed, as seen in all SEM images. Blank paper fibers appeared to have a typical performance for paper. Additionally, the paper coating with the NCS@10 ka/ZnONPs SEM image illustrated a significant appearance of not only the filling of gaps between fibers but also the fiber surface behaviors. In this context, the SEM images for all applied nanocoatings with different ratios of KA/ZnONPs were assigned, whereas the metallic particles were seen in the fiber gaps with different ratios according to nanocoating Ka/ZnONPs. Herein, the fibers of all coated papers were observed coated with transparent and thin coatings. In addition to, the mapping image of Zn ion distribution confirmed that the Zn ion was disturbed homogenously.

#### Antimicrobial study

The nanocoating formulas were tested against six microbial strains, which are representative of the most infectious microorganisms, as shown in Table 2 and comparison with a standrads antimicrobial agents namely; streptomycin as antibacterial and griseofulvin as antifungal. The efficacy of NCS@10 ka/ZnONPs, NCS@20 ka/ZnONPs, and NCS@40 ka/ZnONPs showed an obvious relation between the Ka/ZnONPs percentage contained in the nanocoatings. Moreover, the nanocoating with the highest ratio of Ka/ZnONPs (NCS@40 ka/ZnONPs) showed excellent antimicrobial activity against all presented microorganisms. These results related to broad spectrum antimicrobial activity. Herein, the antimicrobial activity of nanocoating was gained from NCh, Ka, and ZnONPs. Furthermore, the synergetic effect between the three previously mentioned biological active components played a limited role in improving the efficiency of the antimicrobial activity of the prepared nanocoating. In sum, the sample coated with NCS@40 ka/ZnONPs (containing the highest percentage of ZnONPs) was presented an excellent antimicrobial activity against Gram positive and negative bacteria in compared with the standard antibiotic. Otherwise, the effect of NCS@40 ka/ZnONPs against the unicellular fungi in comparison with the standard drug was recorded a close value. These observations were due to the excellent antibacterial activity and moderate antifungal activity of ZnONPs^55–57^.

**Table 2:** Antimicrobial activity of nanocoating formulas against microbial strains.

Samples	Microbes				
	*E. coli*	*P. aeruginosa*	*B. subtilis*	*S. aureus*	*C. albicans*
Blank	0*	0	0	0	0
NCS@10 ka/ZnONPs	41 ± 1.21	32 ± 1.31	53 ± 1.28	29 ± 1.43	22 ± 1.17
NCS@20 ka/ZnONPs	69 ± 1.76	67 ± 1.21	71 ± 1.32	51 ± 1.28	41 ± 1.23
NCS@40 ka/ZnONPs	96 ± 1.55	97 ± 1.34	93 ± 1.67	93 ± 2.23	72 ± 1.58
Streptomycin	71 ± 1.34	79 ± 2.25	51 ± 2.11	56 ± 2.21	NA**
Griseofulvin	NA	NA	NA	NA	68 ± 1.97

*Antimicrobial activity %

**This antibiotic is not applicable for this strain.

### Evaluation of coated paper properties

#### Optical properties

In recent years, the interest in and demand for high-brightness paper has compelled paper manufacturers to devise novel techniques for enhancing the brightness and whiteness of coated paper. Pigment coating is commonly used to improve the optical properties of paper and paperboard, such as brightness, whiteness, and gloss. These optical properties are crucial for the end user and also determine the final price of coated paper^4^. The optical properties of paper products are critical parameters, primarily due to their aesthetic qualities, but they also play a crucial role in print or writing showing through paper products. The properties are defined by reflectance, absorption, and light transmission through paper^34^. Figure 7 represents the optical properties of paper coated with different nanocoatings.

##### Brightness

**Figure 6:**
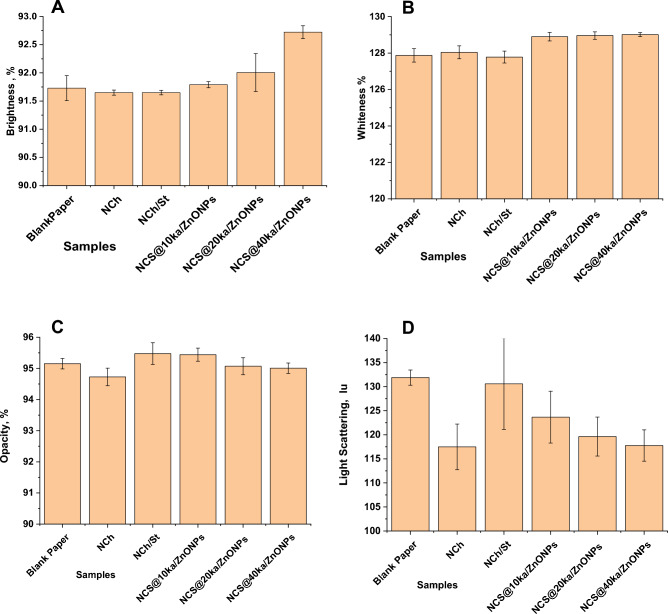
Optical properties of paper coated with various coating suspensions (**A**) Brightness, (**B**) whiteness, (**C**) opacity and (**D**) light scattering.

Coating the blank sample with NCh or NCh/NSt did not affect the paper’s brightness, as shown in Fig. 6A. When Ka/ZnONPs were included in the coating suspensions, the brightness of the coated paper increased slightly. It increased as the percentage of Ka/ZnONPs increased compared to the blank sample. The percentage change reached 1% at the sample NCS@40 ka/ZnONPs.

##### Whiteness

Figure 6B shows that neither NCh nor NCh/NSt suspensions affected the whiteness of coated paper compared to a blank. The addition of Ka/ZnONPs to the coating suspension slightly enhanced the paper’s whiteness. The percentage of increase reached about 1% only when Ka/ZnONPs were loaded with different ratios into NCS.

##### Opacity

Opacity is necessary to prevent printed text from appearing on the back of a sheet of paper. It is directly related to light scattering and the porous coating layer structure^34^. The results in Fig. 6C indicate that coating suspensions containing NCh reduced opacity by approximately 0.45%. In comparison to the opacity of the blank sample, the addition of NCS or NCS@10 ka/ZnONPs resulted in a very slight improvement. The addition of NSt, which is a rheology modifier, increased the viscosity of the solution^5^. Therefore, the opacity of samples containing NCS was increased.

##### Light scattering

NCh-containing coating suspension reduced light scattering by approximately 12%. The addition of NCS to the coating suspension resulted in a 1% decrease in light scattering compared to a blank sample. Figure 6D shows that when Ka/ZnONPs were loaded with different ratios into NCS, light scattering was reduced, and the reduction was proportional to their percentage. The percentage decreased to 6.6, 10, and 12% at the samples NCS@10 ka/ZnONPs, NCS@20 ka/ZnONPs and NCS@40 ka/ZnONPs, respectively, relative to the light scattering of the blank sample.

The particle morphology influences light scattering via the number and size of air microvoids in the sheet. Due to the particle morphology difference between ZnONPs and kaolinite, as the percentage of ka/ZnONPs increased, the air voids in the packing structure of the coating layer decreased, resulting in less light scattering. The size press treatment was implemented to coat paper with ZnONPs using oxidized starch as a binder. ZnONPs were also used in combination with calcined clay to enhance opacity and reduce print through^58^.

#### Mechanical properties

**Figure 7:**
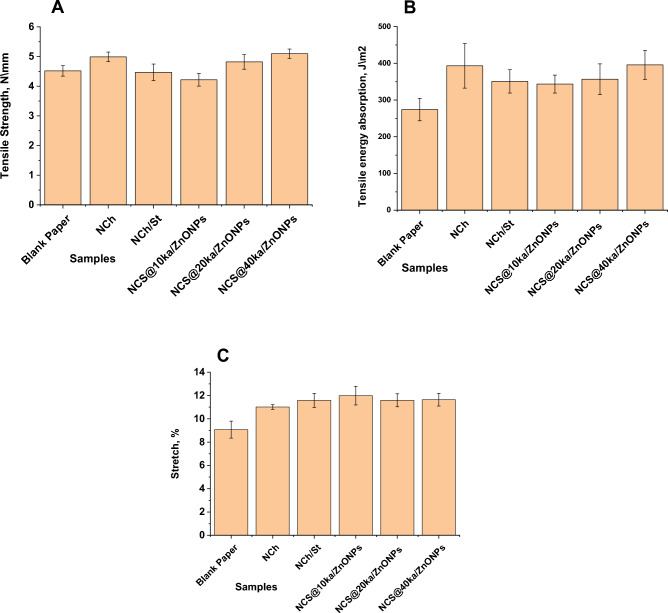
Mechanical properties of paper coated with various coating suspensions (**A**) tensile strength, (**B**) tensile energy absorption and (**C**) stretch.

The mechanical properties of coated paper are important in the examination of the bending and compressive deformation that coated paper undergoes in a printing press, e.g., paper handling and tunability. Figure 7 represents the mechanical properties of paper coated with various coating suspensions.

##### Tensile strength

By introducing NCh to the coating suspension, the tensile strength increased to 9.5%. But by adding NCS, the tensile strength decreased by 1% compared to the blank sample. This decrease continued when the coating suspension contained NCS@10 ka/ZnONPs (Fig. 7A), it reaching 7%. The tensile strength improved as the percentage of ka/ZnONPs increased at the samples NCS@20 ka/ZnONPs and NCS@40 ka/ZnONPs, it reached 6 and 11%, respectively.

##### Tensile energy absorption

Tensile energy absorption (TEA) of all prepared coated paper was increased compared to the blank sample (Fig. 7B). TEA increased by 30.3% by using NCh as a coating suspension. By forming a suspension containing NCS the percentage decreased but was still higher than that of the blank sample by 21.86%. The inclusion of ka/ZnONPs in coating suspension caused increasing TEA by 20%, 23%, and 30.75% of the samples containing NCS@10 ka/ZnONPs, NCS@20 ka/ZnONPs and NCS@40 ka/ZnONPs, respectively.

##### Stretch

The stretch of all prepared coated paper was increased compared to the blank sample, as shown in Fig. 7C. The stretch of samples coated with NCh and NCS increased to 17.63% and 21.65%, respectively. The sample coated with NCS@10 ka/ZnONPs showed the maximum stretch, the increase reached 24.30%. The samples coated with NCS@20 ka/ZnONPs and NCS@40 ka/ZnONPs increased coated paper stretch by 21.7% and 22%, respectively, compared to the blank sample. Chitosan has many applications in the food industry, including antimicrobial film production and coatings. But it has limited mechanical and antimicrobial properties^23^.

Enhancing the antibacterial activity of Ch is achieved by the formation of CS/inorganic composites; thus, the combination of chitosan and ZnONPs develops new biomaterials with excellent antimicrobial activities. This kind of organic–inorganic hybrids not only improve antimicrobial activities, but lower the usage of ZnO and enhance biocompatibility^59^. Nanoclay inclusion in composite films, based on chitosan and nanoclays, led to enhanced mechanical properties. The mechanism of this improvement is related to the formation of intercalated and/or exfoliated composite structures^60–62^.

Nanocomposites exhibit increased barrier properties and mechanical strength compared to their native polymers and conventional composites. Nanocomposites at the nanoscale level result in a large interfacial area, or boundary area, between the biopolymers and nanoparticles. The large interface enabled the modification of molecular mobility and relaxation behavior, as well as the mechanical, thermal, and barrier properties of bio-nanocomposites. The increase in mechanical properties of bio-nanocomposite materials is due to the high rigidity of nanofillers as well as the excellent affinity between biopolymer and nanofiller at the interface^63^.

#### Physical properties

##### Roughness

Roughness describes the topography of the paper surface. It should be low to attain good printing properties. Coated paper roughness is affected by many factors, one of which is the morphology of the pigment particles.

**Figure 8:**
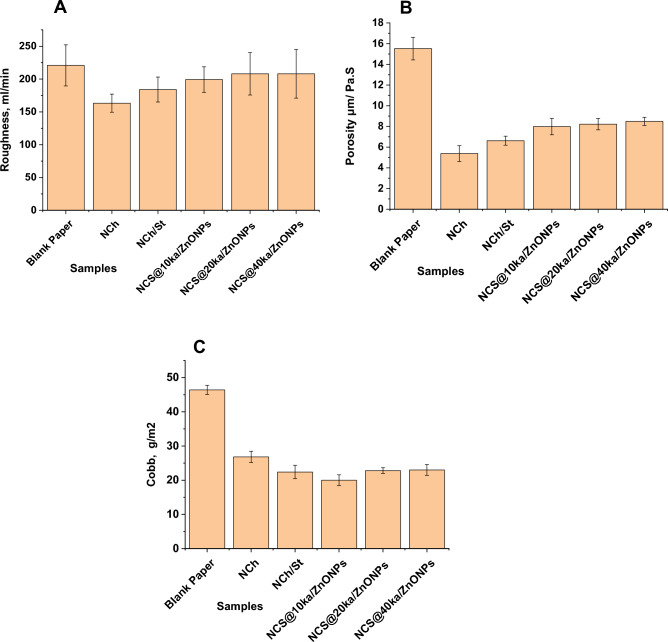
Physical properties of paper coated with various coating suspensions (**A**) roughness, (**B**) porosity, and (**C**) Cobb tests.

Figure 8A represents the effect of different coating suspensions on coated paper roughness. All coating suspensions caused a decrease in coated paper roughness compared to the blank sample. The roughness of paper coated with NCh suspension decreased by 35.32%. In the case of NCS, it decreased by 20.0%. ka/ZnONPs in coating suspension had less effect on coated paper roughness. NCS@10 ka/ZnONPs decreased the roughness by 10.85%, while NCS@20 ka/ZnONPs and NCS@40 ka/ZnONPs decreased the roughness by about 6%.

##### Porosity

Figure 8B represents the results of porosity measurements of paper coated with various coating suspensions. As with roughness, all coating suspensions caused a decrease in coated paper porosity compared to the blank sample. The greatest effect was shown by NCh suspension, which decreased the porosity by 188.6%. NCS decreased it by 134%. The porosity of paper coated with the samples NCS@10 ka/ZnONPs, NCS@20 ka/ZnONPs and NCS@40 ka/ZnONPs decreased by percentages equal to 94.42%, 88.8%, and 82.85%, respectively.

During the coating application, the water leaks into the base sheet. As water is removed from a coating layer, the solid content increases, and a filter-cake layer begins to form on the paper web. When the coating film is set down uniformly to the paper web, the porosity and paper roughness decrease, in other words, the smoothness of the paper increases. The inherent platy structure of kaolinite pigment produces a dense and compact coat layer structure^34^. This high packing characteristic of clay pigment decreased upon increasing the percentage of ZnONPs. Consequently, paper roughness and porosity increased by increasing the percentage of ZnONPs in the combination of ka/ZnONPs.

##### Water absorptivity

Figure 8C represents the water absorptivity of paper coated with various coating suspensions. It was measured by the Cobb test. Water absorbency is a complex and dynamic phenomenon that is influenced by many physical, chemical, and morphological aspects of cellulosic fibers (e.g., surface composition, surface roughness, bulk composition, charged groups, and fiber web porosity^64^. The Cobb value indicates the ability of paper to absorb water. Small Cobb values mean high water resistance in the paper. There is a decrease in Cobb values of all the coated paper compared to the blank paper sample. The minimum decrease in water absorptivity was obtained by coating the paper with NCh suspension. It reached 73%. The maximum decrease was achieved by coating with the sample NCS@10 ka/ZnONPs, the reduction percentage reached 132%. The decrease attained by coating the paper with NCS was 107%. By increasing the percentage of ka/ZnONPs, the Cobb value increased but was still lower than that of the blank paper sample. The samples coated with the suspensions containing NCS@20 ka/ZnONPs and NCS@40 ka/ZnONPs achieved decreases in Cobb values of 104% and 101.7%, respectively.

## Conclusion

Egyptian kaolinite doped ZnONP was loaded onto nanochitosan and nanostarch to formulate a green nanocoating for multifunction paper. This nanocoating was supported by excellent physical, mechanical, optical, and antimicrobial capabilities. Then, these formulas performed antimicrobial activity against six microorganisms that are representative of most poison microorganism families, and the sample containing the highest percentage of ka/ZnONPs represented excellent antimicrobial activity with an inhibition percentage of more than 70% against all tested microorganisms. The properties of coated paper were improved in comparison to blank paper when Ka/ZnONPs were put into NCS in various ratios. The sample NCS@40 ka/ZnONPs improved brightness and whiteness by 1%, decreased light scattering by 12%, and increased tensile strength by 11%. NCh suspension-coated paper showed 188.6% less porosity and 35.32% reduced roughness. Loading of Ka/ZnONPs in NCS reduced porosity by 94% and roughness by 10%. Coating with the sample NCS@10 ka/ZnONPs led to the greatest reduction in water absorptivity, with a reduction percentage of 132%. Finally, the nanocoating formulas successfully combine the development of antimicrobial properties with the enhancement of the qualities of paper, which are crucial for using paper for printing and packaging.

## Data Availability

The data and materials were mentioned in the manuscript and the data was available upon request from the corresponding author.
